# Mitigation strategies for airborne disease transmission in orchestras using computational fluid dynamics

**DOI:** 10.1126/sciadv.abg4511

**Published:** 2021-06-23

**Authors:** Hayden A. Hedworth, Mokbel Karam, Josh McConnell, James C. Sutherland, Tony Saad

**Affiliations:** Department of Chemical Engineering, University of Utah Salt Lake City, UT 84112, USA.

## Abstract

The COVID-19 pandemic forced performing arts groups to cancel shows and entire seasons due to safety concerns for the audience and performers. It is unclear to what extent aerosols generated by wind instruments contribute to exposure because their fate is dependent on the airflow onstage. We use transient, second-order accurate computational fluid dynamics (CFD) simulations and quantitative microbial risk assessment to estimate aerosol concentrations and the associated risk and assess strategies to mitigate exposure in two distinct concert venues. Mitigation strategies involved rearranging musicians and altering the airflow by changing HVAC settings, opening doors, and introducing flow-directing geometries. Our results indicate that the proposed mitigation strategies can reduce aerosol concentrations in the breathing zone by a factor of 100, corresponding to a similar decrease in the probability of infection.

## Teaser

Beyond the six-foot rule: Airflow modification is critical in reducing viral transmission risk among musicians.

## INTRODUCTION

On 10 March 2020, what was supposed to be a routine choir practice for the Skagit County Choir resulted in the infection of 53 of the 61 choir members in attendance with the virus known as severe acute respiratory syndrome coronavirus 2 (SARS-CoV-2), which causes coronavirus disease (COVID-19) (*1*). Three of those infected were hospitalized, and two died (*1*). Further investigation revealed that these infections were due to a single infected person in the choir.

Because of the major uncertainty and risks to public health caused by the COVID-19 pandemic, businesses across the world have been forced to shut down. In particular, the performing arts have suffered immense losses during the pandemic. According to a Brookings Institute report that examined the impact of the COVID-19 pandemic on the arts in the United States, the creative industry affected the most was the fine and performing arts such as choirs, orchestras, operas, and dance companies (*2*). Estimated losses for that sector were a staggering 42.5 billion dollars in lost sales, and more than 50% of jobs lost totaling more than 1.3 million jobs, with even higher estimates worldwide. In the United States, at the time of writing, only a handful of orchestras have returned to in-person performances with limited audiences and many have switched to virtual performances until at least mid-2021.

These statistics are not surprising, given that the vehicle for airborne transmission of infectious diseases, such as COVID-19 and influenza, is primarily exhaled liquid droplets or their dried nuclei (*3*). According to a recent study, the spread of aerosols carried by directional airflow can reach more than 2 m, and COVID-19 transmission may occur in the following methods: (i) through infectious aerosols generated by sneezing, coughing, singing, talking, or playing wind instruments, and (ii) through direct contact with contaminated surfaces or body parts (*4*). These findings highlight the importance of the airflow dynamics in transmitting infectious disease in specific environments.

Computational fluid dynamics (CFD) is an effective tool in characterizing the transport of virally loaded aerosols. CFD has been particularly useful in modeling the spread of disease in environments where general social distancing standards are difficult to maintain, including cars, buses, aircraft cabins, and hospital rooms (*5*–*10*). The infectious aerosols in these and other recent studies are generated by simulating breathing, coughs, and sneezes (*11*–*13*). While these are certainly primary means by which infectious aerosols are produced and spread during day-to-day life, other activities such as singing and playing wind instruments also have the potential to spread diseases through infectious aerosols generated. In the case of wind instruments, data quantifying the size and concentration of aerosols generated have not been reported until recently (*14*). There is also a lack of studies investigating the potential for transmission of disease between wind instrument players in performing groups and in large spaces such as on the stage of a concert hall.

Using data from (*14*) and information about the flow rates from different wind instruments (*15*), we aim to answer the questions: (i) to what extent do wind instruments contribute to the spread of potentially infectious aerosols on stage, and (ii) what mitigation strategies can be used to reduce exposure on stage and decrease the risk of infection? To answer these questions, a locally developed CFD code (*16*) is used to simulate the airflow and spread of fine aerosols (<5 μm diameter) from multiple wind instruments in Abravanel Hall and Capitol Theater, two concert venues both located in Utah. In addition, quantitative microbial risk assessment is used to translate a reduction in aerosol concentration to a reduction in risk of infection. In each venue, we consider the stage area only and are interested in assessing the exposure and risk for the musicians on stage, not the audience.

Abravanel Hall is home to the Utah Symphony, and performances generally include the full orchestra consisting of about 85 instrumentalists. The front of the stage in Abravanel Hall is approximately 24.5 m wide and 10.5 m high. The back of the stage is approximately 14.5 m wide and 7.5 m high, and the stage depth is 12 m. There are two main doors on the stage each around 2.5 m wide and 2.2 m high. There are five rows of supply vents that run along the ceiling and 14 return vents on the floor at the back of the stage. The HVAC (heating, ventilation, and air conditioning) system is designed to supply 240 m^3^/min of air to the stage. Capitol Theater is used for a variety of shows including concerts, operas, plays, and dance performances. The stage is approximately 29 m wide, 16 m deep, and 7 m high. A total of eight air supply vents are located on the left and right sides of the stage, which supply a total of approximately 550 m^3^/min of air to the stage. There is a single return vent at the front of the stage and two doors that are elevated approximately 3 m off the ground along the back wall.

**Fig. 1:**
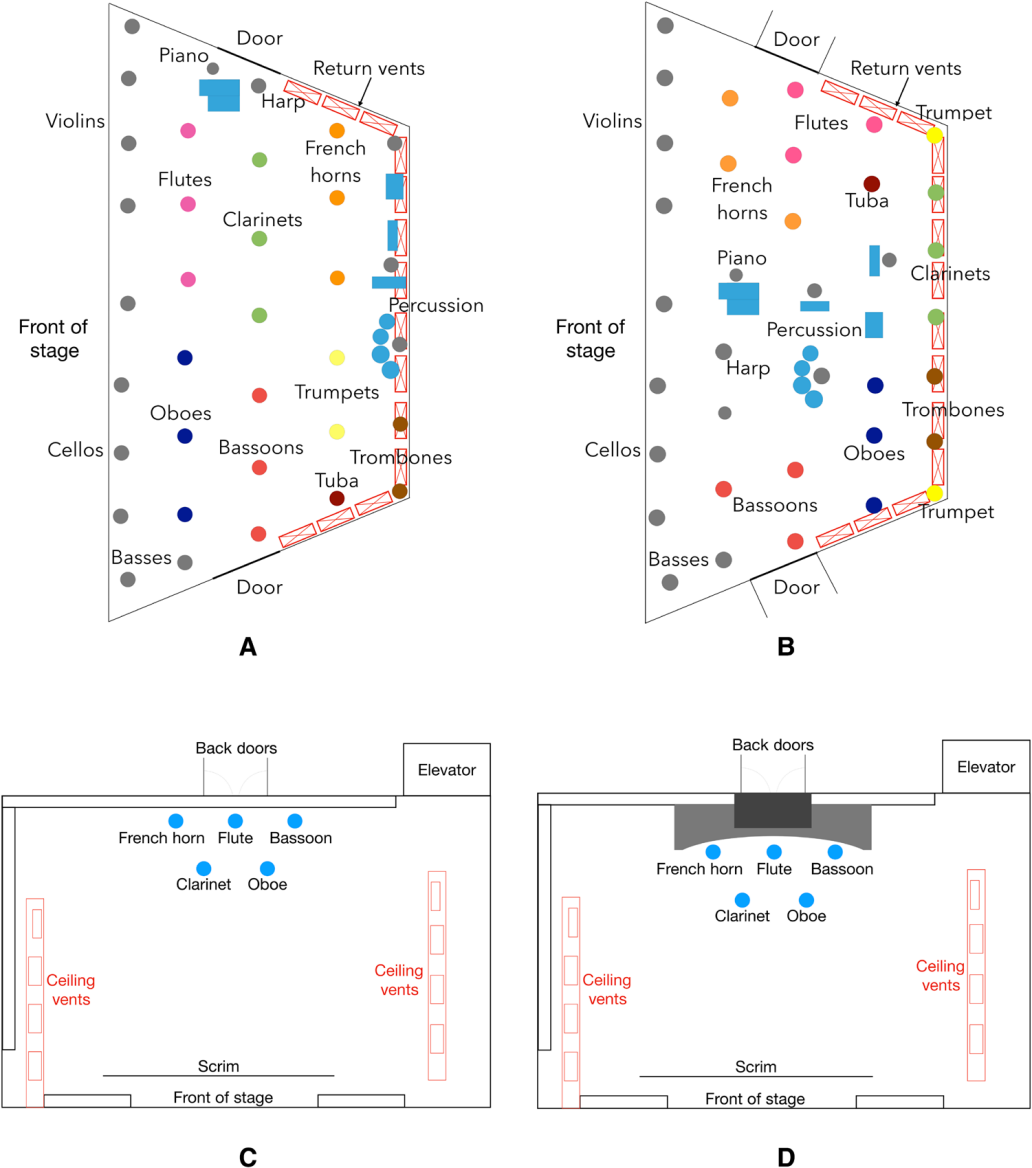
Musician locations on stage for each of the scenarios for both venues. (**A**) Seating arrangement for scenario AH-0. (**B**) Improved arrangement for scenarios AH-1 and AH-2. In Abravanel Hall, some of the string instruments were seated on an extension of the stage that protrudes into the audience area and is not depicted here. (**C**) Seating arrangement for scenario CT-0 and CT-1. (**D**) Arrangement showing the plenum placed over the back doors. In Capitol Theater, string instruments are not depicted but would generally be placed in front of the wind instruments.

The mitigation strategies we consider include rearranging players on the stage and altering the airflow patterns by opening doors and introducing flow-directing geometry. Table 1 summarizes the different scenarios considered for each concert venue. The baseline cases with no mitigation strategies implemented, abbreviated as AH-0 and CT-0, are seating arrangements that were provided by the Utah Symphony and follow local regulations with players at least 2 m apart from each other. The players’ locations for each seating arrangement in both concert halls are shown in Fig. 1. To evaluate the effectiveness of our mitigation strategies, we analyzed the reduction in average aerosol concentration in the region between 0.9 and 1.3 m in height, which corresponds to the breathing zone where the players’ heads are located.

**Table 1:** Mitigation strategies for each of the scenarios considered. Cases AH-0 and CT-0 are the baseline cases that use seating arrangements provided by the Utah Symphony and Utah Opera.

**Identifier**	**Venue**	**Mitigation strategy**
AH-0	Abravanel Hall	None
AH-1	Abravanel Hall	Musician arrangement
AH-2	Abravanel Hall	Musician arrangement + side doors opened
CT-0	Capitol Theater	None
CT-1	Capitol Theater	Rear doors open
CT-2	Capitol Theater	Rear doors open with plenum

For each venue, the airflow pattern is reported first followed by the breathing zone concentrations with different mitigation strategies applied. Then, the effectiveness of each mitigation strategy is discussed, as well as the applicability of our strategies to other venues. Last, limitations of the current study are presented with potential improvement for future work.

## RESULTS AND DISCUSSION

### Abravanel Hall airflow

**Fig. 2:**
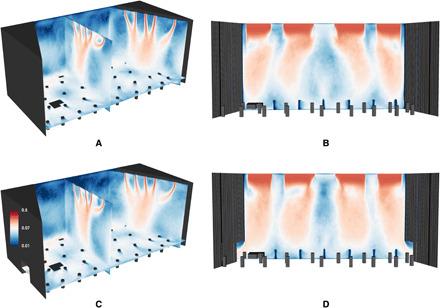
Time-averaged velocity field in Abravanel Hall. (**A**) Slices at center stage and stage left with doors closed. (**B**) Front view of the velocity at mid-stage with doors closed. (**C**). Slices at center stage and stage left with doors open. (**D**) Front view of the velocity at mid-stage with doors open. The colormap ranges from 0.01 to 0.5 m/s on a log scale.

Figure 2 shows contours of the velocity averaged over a 30-min period starting at a residence time of 10 min for scenario AH-0 and a scenario with the same instrument arrangement as AH-0 but with the doors open. The airflow from the ceiling converges inward toward the center row of vents and impinges near the midpoint of the stage, as shown in Fig. 2 (A and C). This forms several large-scale vortical structures: one along the back wall and another weaker one near the front of the stage. These structures can entrain emissions and recirculate them, allowing opportunities for emissions to build up overtime. Figure 2B shows a front view slice of the velocity field at mid-stage, with the flow coming down from the vents. There is a recirculation created in the center of the stage with the flow impinging to the floor and curling back upward where it is then entrained back into the downward flow, creating a recirculation zone where emissions can build up over time. Figure 2D also shows the velocity field at mid-stage and shows that opening the doors weakens the recirculation in the middle of the stage. On the basis of this information about the airflow and vortical structures on stage where recirculation occurs, we were able to effectively rearrange where instruments were located on stage to minimize accumulation of emissions. We placed non-emitting instruments, such as the piano and percussion, in the center of the stage and placed bassoons, which emit higher in the air, near the doors. All other instruments were placed as close to a vent as possible, prioritizing instruments with higher emission rates.

### Abravanel Hall aerosol concentration

**Fig. 3:**
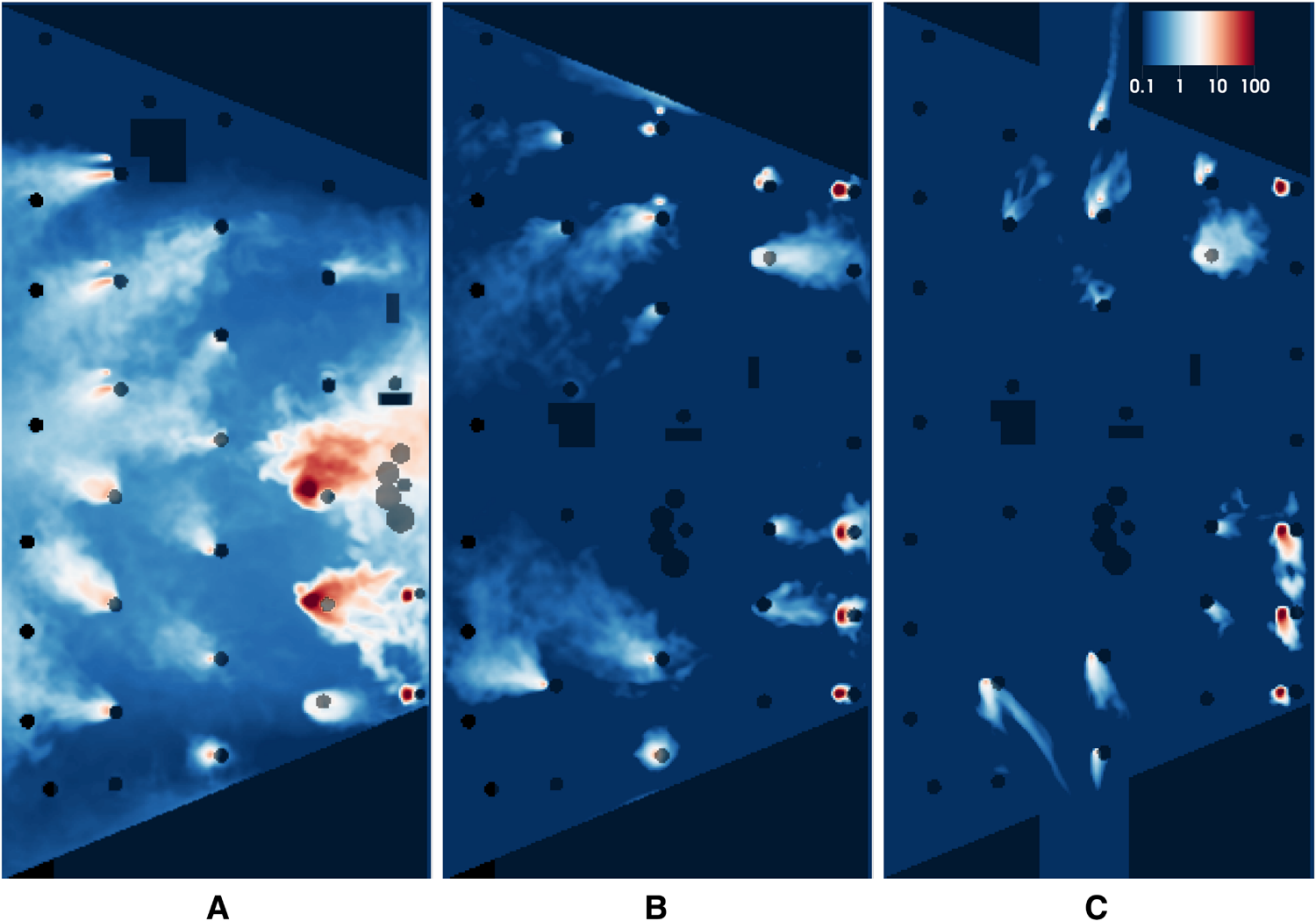
Aerosol concentrations in the breathing zone in Abravanel Hall. The concentrations were averaged over a time span of 20 min and height ranging from 0.9 to 1.3 m for scenarios AH-0 (**A**), AH-1 (**B**), and AH-2 (**C**). The colormap ranges from 0.1 to 100 particles per liter on a log scale.

Figure 3 shows contours of aerosol concentration (on a logarithmic scale) averaged over a time span of 20 min, starting at a simulation time of 8 min. This time interval corresponds to four residence times because the average residence time in Abravanel Hall is approximately 5 min. The concentration was also averaged spatially over a height ranging from 0.9 to 1.3 m, which corresponds to the breathing zone for the musicians while seated. As illustrated by the concentration contours for the original arrangement, AH-0 (Fig. 3A), the mean aerosol concentration over much of the stage area approaches or exceeds 5 particles per liter. Large, high-concentration (>20 particles per liter) plumes are evident at the rear of the stage where the trumpets are located. Moving the high-emission instruments near the return vents along the walls of the stage substantially reduces the aerosol concentration, as indicated by the concentration contours for case AH-1 (Fig. 3B). However, sizable plumes of approximately 1 particle per liter are present, particularly around the woodwind instruments not immediately adjacent to a return vent. Figure 3C shows results when the doors at the sides of the stage are opened. This scenario results in virtually no accumulation of emissions from the woodwind instruments and very minimal infringement on other instrumentalists’ breathing zones.

**Fig. 4:**
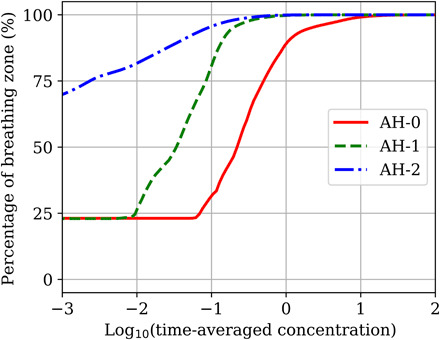
Cumulative function for percentage of the breathing zone volume in Abravanel Hall occupied by a certain concentration range. The *x* axis is plotted on a log scale. The *y* axis values represent the percentage of the breathing zone with a concentration less than or equal to the corresponding *x* axis value.

Figure 4 shows an analysis of the breathing zone based on the time-averaged concentration in each individual grid cell. The *y* axis shows the percentage of the volume in that region that is occupied by concentrations less than or equal to the corresponding value on the *x* axis. A cylindrical region of radius 0.75 m around each instrumentalist was ignored to remove emission sources. The concentration is averaged over a 10- to 30-min interval starting at a simulation time of 10 min but not spatially averaged. Figure 4 shows that, in scenario AH-0, approximately 70% of the volume of the breathing zone is occupied by concentrations between 0.1 and 10 particles per liter. The rearranged seating in scenario AH-1 reduces the buildup of emissions by approximately one order of magnitude. For that scenario, 70% of the breathing zone has a concentration between 0.01 and 1 particle per liter. Opening the doors in addition to rearranging the instruments provides a large reduction in the accumulation of emissions where 70% of the domain is occupied by concentrations of less than 0.001 particle per liter. Given the uncertainty in our concentrations due to the various assumptions that were necessary, and the uncertainty in the dose-response model applied, absolute probabilities of infection are not reported. However, valuable insight can still be gained by applying the dose-response model and discussing relative reductions in infection probability. Because concentration and dose are linearly related, a reduction in concentration by two to three orders of magnitude results in the same reduction in the dose. For doses less than 100 plaque-forming units (PFU), the probability of infection is also linearly related to the dose. The doses calculated from our simulations all fell into this range, meaning that for a two to three order of magnitude reduction in the concentration, the probability also decreases by approximately the same amount. These results demonstrate the importance of analyzing the airflow and providing sufficient ventilation in indoor environments where exposure to airborne pathogens is a concern. In addition to social distancing, simple strategies like opening doors can be an effective way to decrease the concentration of infectious aerosols and the risk of infection.

### Capitol Theater airflow

**Fig. 5:**
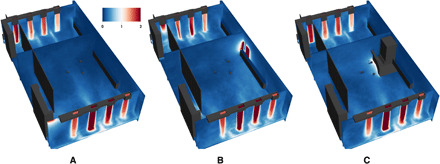
Time-averaged velocity field in Capitol Theater. Slices of the time-averaged velocity field at the level of the inlet vents, the level of the players, and mid-stage where the doors are located for scenarios CT-0 (**A**), CT-1 (**B**), and CT-2 (**C**). The color bar ranges from 0 m/s (dark blue) to 1 m/s (white) up to 2 m/s (dark red) on a linear scale.

Figure 5 shows slices of the time-averaged velocity field for each of the scenarios in Capitol Theater. The velocity was averaged over a 10-min period starting at a residence time of 10 min. The flow in the middle of the stage around the players is relatively low (∼0 to 0.25 m/s), and there are no large vortical structures or recirculations as in Abravanel Hall. Even when the doors at the back of the stage are opened, there is little change in the airflow at the level of the players, as shown in Fig. 4, due to doors being elevated about 3 m off the stage. Because of the lack of airflow around the players, it was necessary to alter the flow pattern on stage using additional geometry to create a path from the players to an outlet. Figure 4 shows that building a plenum over the back doors that extends down to the level of the players creates a stronger flow (∼1 m/s) directly behind the players and provides an effective means of flushing out emissions from the instruments.

### Capitol Theater aerosol concentration

**Fig. 6:**
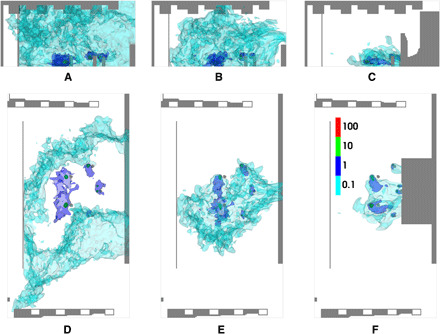
Iso-contours of the time-averaged aerosol concentration at Capitol Theater. (**A** to **C**) View from the stage left side for scenarios CT-0, CT-1, and CT-2, respectively. (**D** to **F**) View from above the stage for scenarios CT-0, CT-1, and CT-2, respectively. Contours range from 0.1 to 100 particles per liter on a log scale.

Figure 6 shows views from the top and side of the domain of the concentration plume averaged over a 10-min period for each of the three scenarios. While there are fewer wind instruments present in Capitol Theater, the suboptimal HVAC system is not able to circulate air through the domain and flush out emissions from the instruments. The plume of emissions in scenario CT-0 (Fig. 6, A and D) spreads through the entire height of the domain and into the space in front of the wind instruments where string players and other musicians would sit. Scenario CT-1 has the same arrangement of wind instruments as CT-0, but the doors located on the back wall are opened in an attempt to reduce the emission plume. As shown in Fig. 6 (B and E), opening the doors reduces the width of the plume, but due to the doors being elevated above the level of the instruments, this strategy is not adequate to clear emissions from the area around and in front of the wind instruments. To use the open doors as a means of clearing emissions, another scenario, CT-2, was considered with a plenum added that covers the doors and extends down to the level of the players. A shell structure similar to those used for choirs was also added around the instruments. This added geometry creates an air return directly behind the wind instruments. The plume in scenario CT-2 (Fig. 6, C and F) is greatly reduced in size, and the emissions are quickly directed through the plenum and out the back doors. In each scenario, the plumes range in concentration between 0.1 and 1 particle per liter, with some higher concentrations (≥10 particles per liter) located at or near the outlet of the instruments.

**Fig. 7:**
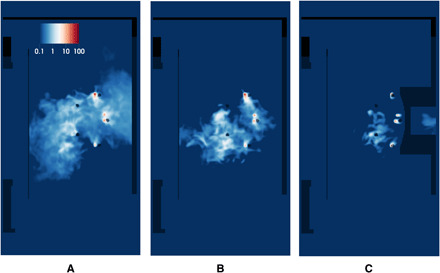
Aerosol concentrations in the breathing zone of Capitol Theater. The concentration is averaged over a time span of 20 min and height ranging from 0.9 to 1.3 m for scenarios CT-0 (**A**), CT-1 (**B**), and CT-2 (**C**). The colormap ranges from 0.1 to 100 particles per liter on a log scale.

Figure 7 shows the concentration of aerosol particles emitted from the five instruments averaged over time and over a region of the domain from a height of 0.9 to 1.3 m. For scenario CT-0 (Fig. 6), the suboptimal conditions of the HVAC system allow emissions to spread throughout the domain and accumulate in the region between the scrim and the back wall where other instrumentalists would be seated. The concentrations that reach the front of the scrim are about an order of magnitude (≈0.1 to 1 particle per liter) lower than the concentrations near the wind instruments (≈1 to 10 particles per liter). This scenario emphasizes the fact that airflow analysis is essential in characterizing the fate of emissions and the potential for exposure. Although there are only five wind instruments, the mixing and lack of ventilation allow the aerosol particles to spread and accumulate throughout the main stage area. In Capitol Theater and similar venues, preventative measures like making adjustments to the ventilation system may be necessary in addition to social distancing and wearing masks, especially because it is impractical for some musicians to wear a mask while playing. Opening the rear doors, as shown in Fig. 7B, reduces the distance over which emissions travel. The plume remains in the center of the stage area and barely reaches the scrim. The addition of the plenum over the doors further reduces the spread of emissions, as shown in Fig. 7C, and keeps the plume concentrated in the area between the players and the shell.

**Fig. 8:**
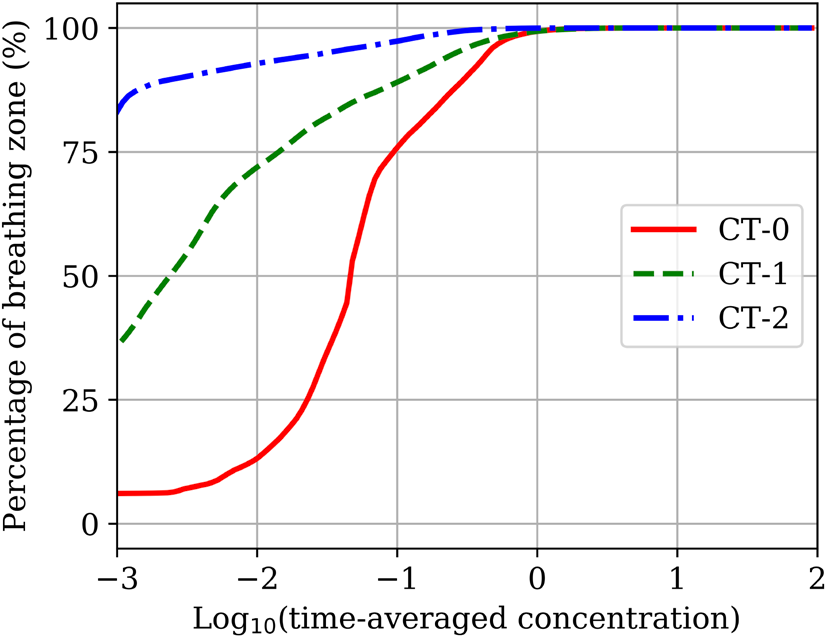
Cumulative function for percentage of the breathing zone volume in Capitol Theater occupied by a certain concentration range. The *x* axis is plotted on a log scale. The *y* axis values represent the percentage of the breathing zone with a concentration less than or equal to the corresponding *x* axis value.

The breathing zone in Capitol Theater was analyzed by individual grid cells using the same method as Abravanel Hall. Figure 8 shows the percentage of the domain occupied by concentrations less than or equal to the corresponding value on the *x* axis. For scenario CT-0 with no mitigation, the concentration in 75% of the breathing zone is between 0.02 and 1 particle per liter. When the rear doors were opened, there was a reduction in the spread of emissions and only 20% of the breathing zone had concentrations within the range between 0.02 and 1 particle per liter. A considerable portion of the breathing zone (≈35%) had a concentration of 0.001 particle per liter or less. The addition of the plenum notably decreased the spread of emissions. Less than 6% of the breathing zone had concentrations between 0.02 and 1 particle per liter, and 83% of the breathing zone had a concentration below 0.001 particle per liter. Similar to Abravanel Hall, there is an approximately linear relationship between the concentrations observed in Capitol Theater, the doses, and the infection probabilities. Thus, the reduction in concentration by about two orders of magnitude achieved by opening the back doors and installing the plenum corresponds to a reduction in dose and probability of about two orders of magnitude. Airflow analysis of Capitol Theater showed that using existing air returns and doors to ventilate the stage area was not possible and that, without adding flow-altering geometry, it would be difficult to remove emitted aerosol particles effectively. While the strategies we implemented worked well for the chosen arrangement of instruments, their effectiveness would likely decrease with a larger group of wind instruments or a performance that required musicians to be seated farther from the doors. The challenges associated with Capitol Theater are likely characteristic of other indoor venues with older HVAC systems or poor ventilation. While airflow will differ from building to building, analysis of the airflow is a critical first step to make informed decisions on which mitigation strategies to apply.

### Limitations and future work

There are several limitations of our approach to model the emissions from wind instruments. Because some of the instruments’ bell sizes were smaller than the grid resolution, we modeled them as having larger bell sizes and lower velocities to maintain a consistent volumetric flow rate. This changes the local flow field at the instrument outlet and around the player and could affect the quantity of the aerosol particles that reach other adjacent players. Our model also neglected the effects of temperature variations, relative humidity, and buoyancy on the plumes emitted from the instruments. These factors can affect the size and transport of aerosol particles as well as viral viability. Despite these limitations, this work provides insight into the contributions of wind instruments in spreading potentially infectious aerosols to other players during a performance and provides valuable analysis of strategies that can be easily implemented to mitigate the spread of aerosols and allow safer performances.

The limitations in this study stem from the challenging nature of modeling complex transport phenomena in a domain as large as a concert hall and the time frame allotted for the project (∼10 weeks). Improvements such as refining the computational mesh further to capture the flow from smaller instruments, modeling the evolution of the particle size distribution, and improving boundary condition characterization would certainly improve the accuracy of the results. However, this comes with an increased cost in terms of both computational time and resources. Detailed CFD studies such as these provide valuable insight but remain intractable for many organizations. Simple alternatives that can provide basic insight into large-scale fluid flow patterns and some indication of the efficacy of proposed mitigation strategies include usage of common equipment such as fog machines.

## METHODS

The unsteady Navier-Stokes equations are the governing equations we use to simulate airflow dynamics. To model turbulence, we use a large eddy simulation (LES) methodology. LES enables us to capture the highly transient features of the airflow created by the HVAC and its interaction with the wind instruments. It is also justified by the fact that grid resolution is small enough (∼5 to 10 cm) to resolve the salient features of the airflow. Momentum conservation is achieved by solving the Favre-filtered Navier-Stokes equations

$$\rho \frac{\partial \bar{u}}{\partial t}=-\rho \nabla \cdot (\bar{u}\bar{u})-\nabla \bar{p}-\nabla \cdot {\bar{\tau }}_{\text{ij}}-\nabla \cdot \tau^{R}$$ (1)

$$\nabla \cdot \bar{u}=0$$ (2)

where ρ is the density (assumed to be constant) and $\bar{u}$, $\bar{p}$, and $\bar{\tau }$ are the resolved (filtered) velocity, pressure, and stress tensor, respectively. The stress tensor is given in terms of the rate of strain tensor ${\bar{\tau }}_{\text{ij}}=2\mu {\bar{S}}_{\text{ij}}=\mu (\frac{\partial {\bar{u}}_{i}}{\partial x_{j}}+\frac{\partial {\bar{u}}_{j}}{\partial x_{i}})$, where μ is the dynamic viscosity of the fluid. The quantity **τ**^R^ is the unresolved or subgrid stress tensor and requires modeling. To calculate **τ**^R^, we use an eddy viscosity model where $\tau_{\text{ij}}^{R}=2\mu_{t}{\bar{S}}_{\text{ij}}$, with all turbulence energy cascade effects absorbed into the turbulent viscosity μ_t_. Last, the Smagorinsky-Lilly model is used to compute the turbulent viscosity via

$$\mu_{t}=\rho C_{s}\Delta \sqrt{2{\bar{S}}_{\text{ij}}{\bar{S}}_{\text{ij}}}=\rho C_{s}\Delta |\bar{S}|$$ (3)

where the filter width is related to the grid cell size by $\Delta =V_{\text{cell}}^{1/3}$, *V*_cell_ is the volume of a grid cell, and *C*_s_ is a constant set to 0.2 in our code based on previous verification of our turbulence models.

Aerosol particles from each type of instrument are represented as a unique scalar variable whose spatial and temporal evolution is governed by a transport equation of the form

$$\rho \frac{\partial {\bar{\phi }}_{i}}{\partial t}+\rho \nabla \cdot (\bar{u}{\bar{\phi }}_{i})=(D_{i}+D_{t})\nabla^{2}{\bar{\phi }}_{i}$$ (4)

where ϕ_i_ is the particle concentration, *D_i_* and $D_{t}=\frac{\mu }{\rho {\text{Sc}}_{t}}$ are the molecular and turbulent diffusivities of ϕ_i_, and Sc_t_ = 0.7 is the turbulent Schmidt number. For the scenarios considered, *D_t_* ≫ *D_i_*. The aerosol concentrations emitted by the various wind instruments were obtained from (*14*) and are reported in Table 2 along with the maximum volumetric flow rate of air exiting the instruments (*15*) and the approximate bell radius. Only the outlet of each instrument is represented. Each instrument outlet is represented as a circle with a radius corresponding to a standard bell size for that instrument. Instruments with a bell size smaller than the grid resolution are modeled as having a radius equal to the grid resolution. The velocity of those instruments is scaled based on the numerical area of the instrument outlet such that the volumetric flow rate and aerosol concentration match experimental data (*14*,*15*).

**Table 2:** The aerosol particle concentration and volumetric flow rate at the outlets of different musical instruments.

**Instrument**	**Concentration**	**Volumetric** **flow rate**	**Bell radius**
	(particles/liter)	(liter/min)	(m)
Tuba	18	101	0.25
Bassoon	40	42	0.02
Flute	45	11	0.01
Flautist	45	26	0.005
French horn	90	36	0.15
Clarinet	200	20	0.03
Trombone	300	47	0.11
Oboe	400	9	0.025
Trumpet	2500	28	0.06

The probability of infection is estimated from the aerosol concentrations using the dose-response model *p*(*d*) = 1 − *e*^−*d*/410^, fitted using data for the SARS-CoV virus, where *d* is the dose in PFU (*17*). The number of virus copies per particle was assumed to be constant and estimated as 0.1 virus per particle using data from (*18*) on virus emission rates from individuals infected with COVID-19 and data from (*14*) on particle emission rates from instruments. The dose was calculated using particle concentrations from our simulations, the estimated virus load per particle, an estimated breathing rate of 0.012 m^3^/min (*19*), and an exposure time of 5 min.

During a performance, wind instruments generally have periods of rest where they are not playing and emitting particles into the air. In our approach, however, instruments emit particles continuously throughout the entire simulation at a constant rate. Particles emitted from an instrument have a size distribution, and the size of the particle strongly affects its fate: Large particles quickly settle to the ground, while small particles are entrained with the flow. Small particles also evaporate faster. This study does not seek to track the evolution of particle size distributions. Rather, our approach to computing aerosol particle concentration assumes that all particles are entrained in the flow, and that they are persistent (do not evaporate). Studies evaluating aerosol transport in indoor environments have shown that particles smaller than 10 or 20 μm in diameter can be accurately represented as passive scalars that follow the flow (*20*, *21*). The study that we used to set instrument emission rates found that the mean particle diameter for 10 different instruments was less than 4 μm (*14*). On the basis of their findings, it is reasonable to assume that the emissions from wind instruments considered in this work are all entrained in the flow. We also assumed that the virus concentration in the aerosol particles was uniform and did not account for changes in viral stability or inactivity. Temperature and relative humidity both affect the evaporation of droplets and the stability of viruses within droplets (*12*, *22*, *23*). However, modeling these relationships was beyond the scope of this study. Our assumptions likely result in overestimates of particle emission and therefore provide conservative estimates for aerosol concentration and risk.

For the different simulation scenarios, we used a staggered, uniformly spaced grid. In Abravanel Hall, the cell dimensions were 5 cm × 5 cm × 5 cm and we used a total of 14.6 million cells. In Capitol Theater, the cell dimensions were 10 cm × 10 cm × 10 cm and we used a total of 3.6 million cells. For the spatial discretization, we used a central difference scheme on the momentum equations and the SuperBee flux limiter on the scalar transport equations. The average residence times for Abravanel Hall and Capitol Theater were between 5 and 10 min. Each simulation was run for approximately four to eight residence times. Dirichlet conditions were used on the velocity at the inlet and outlet ducts, where the value of the volumetric flow rate is known. The air entering at the inlet vents was assumed to be clean with no recirculation of aerosols through the HVAC system. Open boundary conditions were used at the locations where the air enters or exits the domain at unknown velocities. We used a forward Euler integrator to advance all equations in time.
